# Supplementary material for: Inhibition of the SUV4-20 H1 histone methyltransferase increases frataxin expression in Friedreich's ataxia patient cells
Source: J Biol Chem. 2021 Jan 13;295(52):17973–85. doi: 10.1074/jbc.RA120.015533 (PMC7939392; doi:10.1074/jbc.RA120.015533)
Supplement: Supplementary file 1 [file mmc1.pdf]

Inhibition of the SUV4-20 H1 histone methyltransferase increases frataxin expression in Friedreich's ataxia patient cells

Vilema-Enríquez G[1], Quinlan R[2,3], Kilfeather P[1], Mazzone R[2,3], Saqlain S[1], del Molino del Barrio I[1], Donato A[1], Corda G[1], Li F[4], Vedadi M[4,5], Németh AH[6,7], Brennan PE[2,3] & Wade-Martins R*[1]

Material included

Primers sequence used in the study, screening conditions used for each probe, toxicity assay (adenylate kinase) of each screened probe, effect of the hit molecules on the *FXN*-Luc cell line, concentration-responses of compounds 4 and 23, down-regulation of *EZH1* and *EZH2* in the *FXN*-GAA-Luc cell line, down-regulation of *DOT1L* in the *FXN*-GAA-Luc cell, list of genes that represent affected pathways in H4K20 mono-methylated state, effect of A-196 on control-derived cells, crystal structure of A-196 bound to SUV4-20 H1, IC_{50} determination of each A-196 analogue using SUV4-20 H1 (summary of the results and activity curves), PCA bi-plot of fibroblasts excluded from the analysis, Chemical structural modification of A-196.

Primers list

FXN

FXN-F (5′-AACGTGGCCTCAACCAGATTT -3′)

FXN-R (5′-GGTGGCCCAAAGTTCCAGATT-3′)

SUV4-20 H1

SUV4-20H1-F (5′-TGCGAATGTTTGCAACTGACA -3′)

SUV4-20H1-R (5′-TTCGGCAATACAACCGACCA-3′)

SUV4-20 H2

SUV4-20H2-F (5′-GAAACTTTCCTGAGGCAGCG -3′)

SUV4-20H2-R (5′-CGGCTCTGGAAGTAGCGG-3′)

HRPT1

HRPT1-F (5′-GCCAGACTTTGTTGGATTTG -3′)

HRPT1-R (5′-CTCTCATCTTAGGCTTTGTATTTTG-3′)

Supplementary Table 1. SGC epigenetic chemical probes library used in this study.

Compound number	Probe's name	Target	Working concentration (μM)	Incubation time
1	(+)-JQ1	BRD2, BRD3, BRD4, BRDT (BET)	1	48 h
2	(R)-PFI-2 (hydrochloride)	SETD7	4	48 h
3	A-196	SUV420H1/H2	4	6 d
4	A-366	G9a, GLP	4	6 d
5	BAY-598	SMYD2	4	48 h
6	BAZ2-ICR	BAZ2A, BAZ2B	4	48 h
7	BI-9564	BRD9, BRD7	4	48 h
8	Bromosporine	pan-Bromodomain	5	48 h
9	CI-994	Class I histone deacetylase inhibitor	Not screened	-
10	GSK2801	BAZ2A, BAZ2B	4	48 h
11	GSK343	EZH1/2	4	6 d
12	GSK484 (hydrochloride)	PAD-4	10	48 h
13	GSK591	PRMT5	4	6 d
14	GSK864	Mutant isocitrate dehydrogenase 1	1	48 h
15	GSK-J1 (hydrochloride)	JMJD3, UTX, JARID1B	5	48 h
16	GSK-LSD1 (hydrochloride)	LSD1	1	48 h
17	I-BRD9	BRD9	4	48 h
18	I-CBP112 (hydrochloride)	CREBBP, EP300	4	48 h
19	IOX1	2-oxoglutarate oxygenases	Not screened	-
20	IOX2	HIF prolyl-hydroxylases	Not screened	-
21	LAQ824	histone acetylase inhibitor	6	48 h
22	LP99	BRD9, BRD7	4	48 h
23	MS023 (HCl salt)	Type I PRMTs	4	6 d
24	MS049 (HCl salt)	PRMT4,6	4	6 d
25	NI-57	BRPF1, BRPF2, BRPF3	4	48 h
26	NVS-CECR2-1	CECR2	1	48 h
27	OF-1	BRPF1, BRPF2, BRPF3	4	48 h
28	OICR-9429	WDR5	4	48 h
29	Olaparib	PARP1, PARP2	1	48 h
30	PFI-1	BRD2, BRD3, BRD4, BRDT (BET)	4	48 h
31	PFI-3	SMARCA,PB1	4	48 h
32	PFI-4	BRPF1B	4	48 h
33	SGC0946	DOT1L	4	6 d
34	SGC707	PRMT3	4	48 h
35	SGC-CBP30	CREBBP, EP300	3	48 h
36	UNC0638	G9a, GLP	4	6 d
37	UNC0642	G9a, GLP	4	6 d
38	UNC1215	L3MBTL3	4	6 d
39	UNC1999	EZH1/2	4	6 d

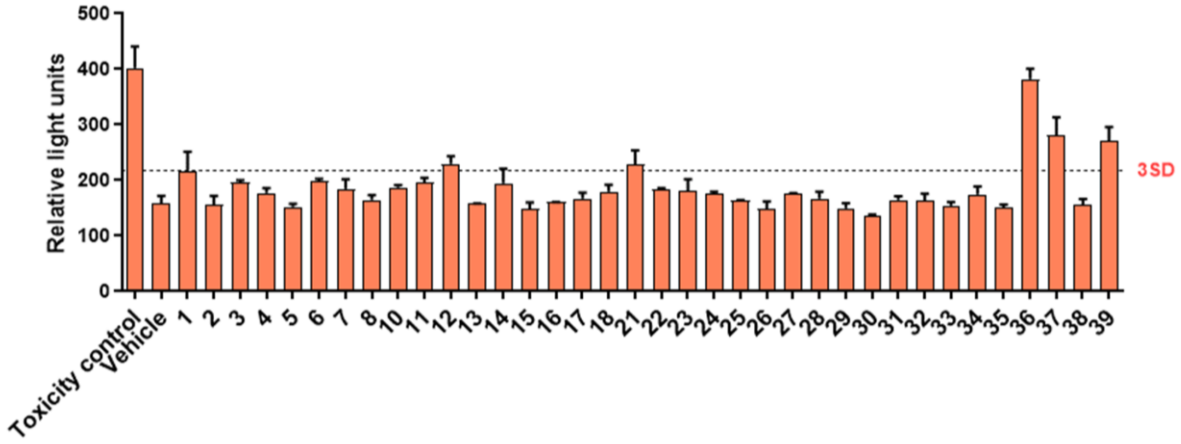

Supplementary Figure 1. Adenylate kinase assay of the *FXN*-GAA-Luc cell line after treatment with the SGC epigenetic probes library

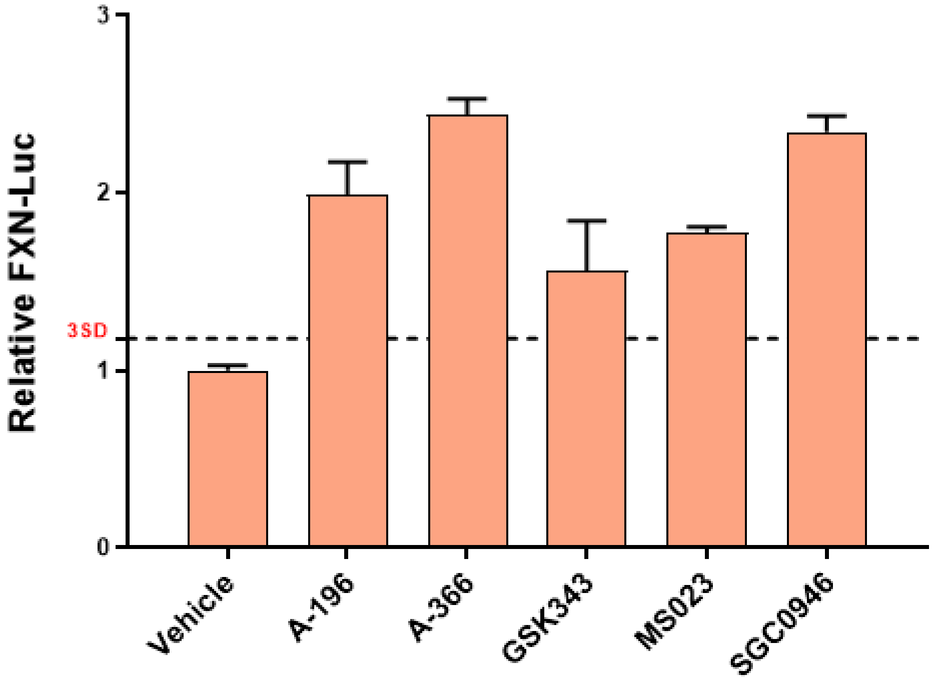

Supplementary Figure 2. Luciferase assay of the *FXN*-Luc cell line treated with the hit compounds of the SGC epigenetic probes collection. Treatment was for six days with 4 µM of each probe.

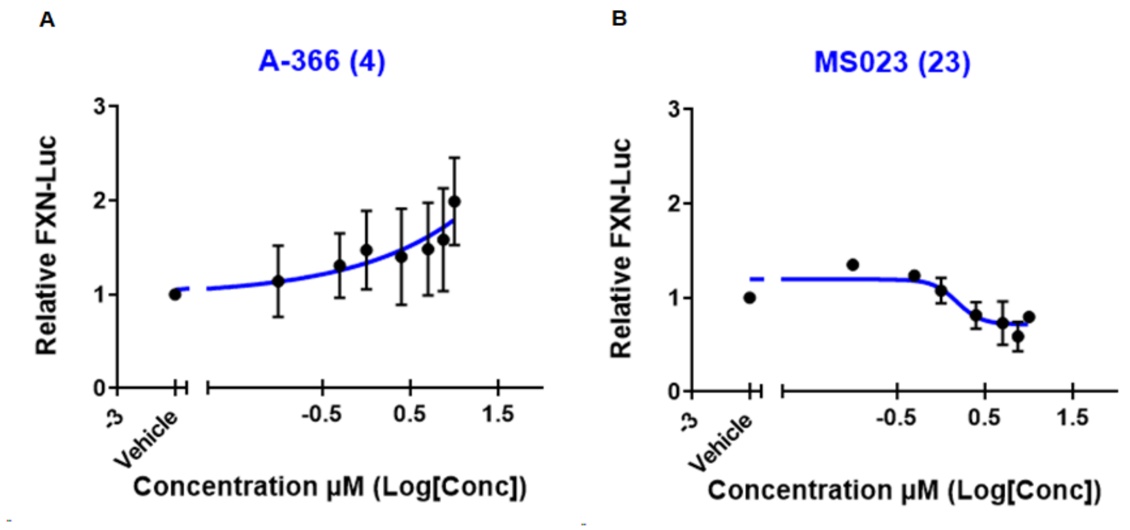

Supplementary Figure 3. Concentration-response curves assessed by luciferase assay of the *FXN*-GAA-Luc cell line treated for six days with **(A)** A-366 and **(B)** MS023.

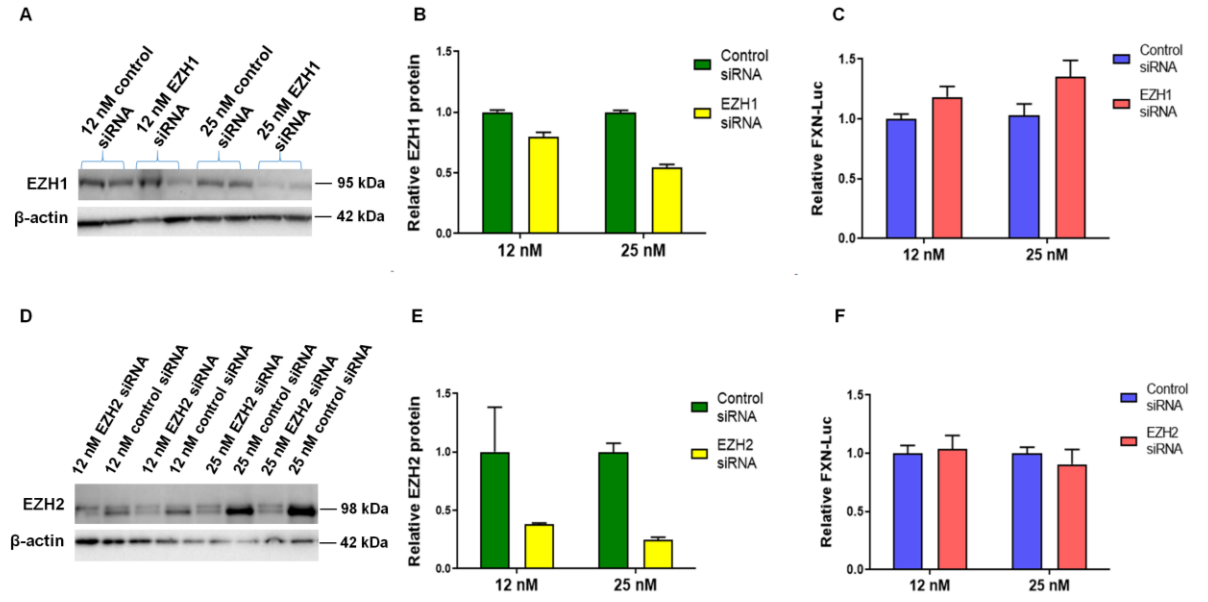

Supplementary Figure 4. Down-regulation of *EZH1* and *EZH2* in the *FXN*-GAA-Luc cell line **(A)** Western blot of EZH1 after siRNA transfection, **(B)** Quantification of panel A corresponding to EZH1 protein after EZH1 siRNA treatment, **(C)** Luciferase assay of *FXN*-GAA-Luc cell line after *EZH1* siRNA-mediated knock-down, **(D)** Western blot of EZH2 after the down-regulation of the protein, **(E)** Quantification of panel D corresponding to EZH2 protein after siRNA treatment, and **(F)** FXN-Luc protein after *EZH2* knock-down, assessed by luciferase assay. Data are presented as mean ± SEM of three independent experiments completed in triplicate, except for the confirmation of knockdowns, which was performed twice in duplicate, two-way ANOVA.

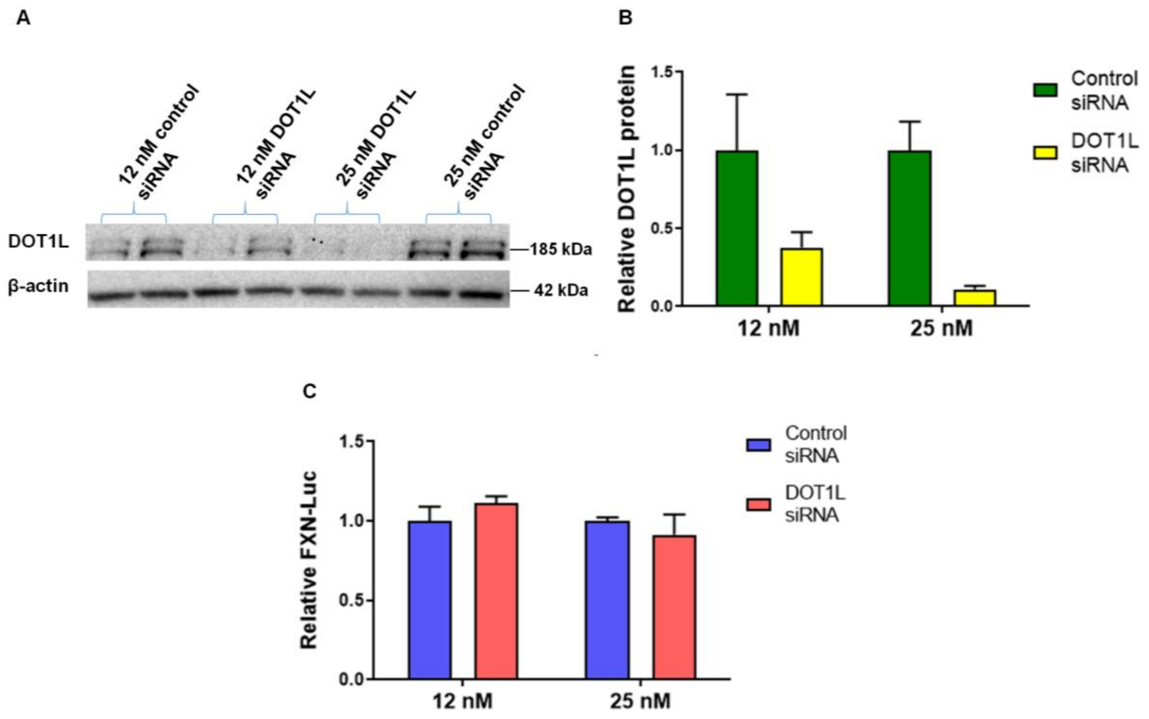

Supplementary Figure 5. Down-regulation of *DOT1L* in the *FXN*-GAA-Luc cell **(A)** Western blot of the *FXN*-GAA-Luc cell line after DOT1L and control siRNA treatment, **(B)** Quantification of panel A corresponding to DOT1L protein after siRNA treatment and **(C)** FXN-Luc protein after down-regulation of DOT1L assessed by luciferase assay. Data are presented as mean ± SEM of three independent experiments completed in triplicate, except for the knock-down evaluation which was performed twice in duplicate, two-way ANOVA.

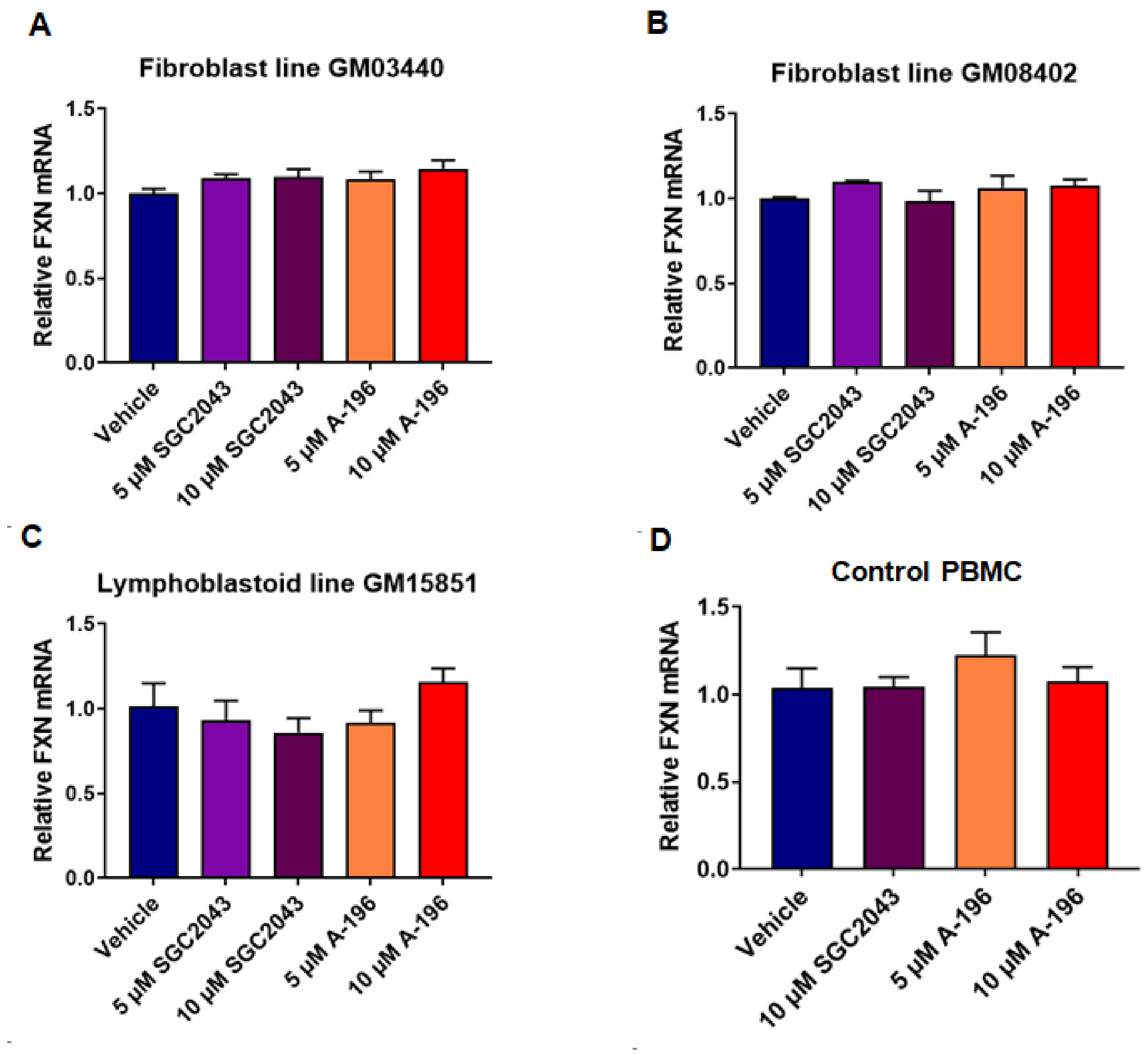

Supplementary Figure 6. A-196 does not increase frataxin mRNA expression in control-derived cells. (A, B) Frataxin qRT-PCR of control-derived primary fibroblast lines GM03440 and GM08402 after A-196 treatment, n=3 (C) Frataxin qRT-PCR of control-derived lymphoblastoid cell line GM15851 after A-196 treatment, n=3 (D) Frataxin mRNA expression after A-196 treatment in PBMCs extracted from three control individuals. Data are relative to the vehicle, a treatment of 6 days and are presented as mean ± SEM, one-way ANOVA followed by Bonferroni test.

Supplementary Table 2: Key genes from Schotta et al., (37) tested for differential expression in A-196-treated samples

**KMT5A**
**KMT5B**
**KMT5C**
**SUV39H1**
**TP53BP1**
**CCNB1**
**CDK1**
**RASGRF1**
**ATM**
**ATR**
**RAG1**
**RAG2**
**MRE11**
**RAD50**
**NBN**
**MDC1**
**AICDA**

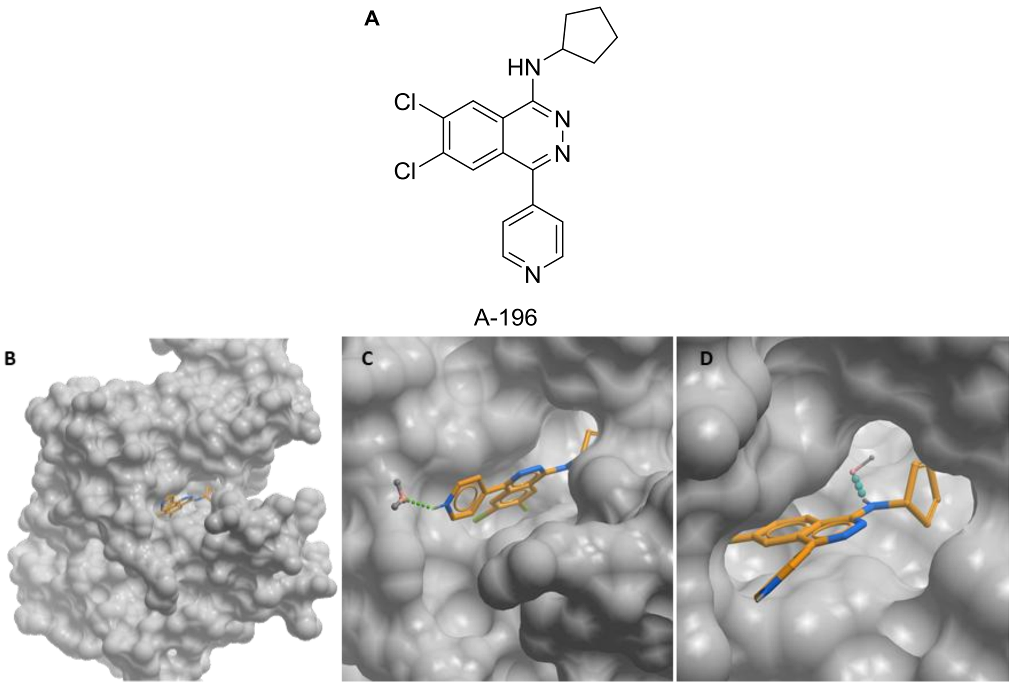

Supplementary Figure 7. A) A-196; B) Crystal structure of A-196 (orange) bound to SUV4-20 H1 (PDB: 5CPR) (ref. 36) C) H-bonding interaction of A-196 pyridyl nitrogen with water; D) Crucial H-bond between A-19 amine N-H and active site water.

Supplementary Table 3. IC_{50} determination using SUV4-20 H1

**Compound**	**SUV4-20H1**	
	**IC_{50}* (μM)**	**Hill Slope**
**Control_A-196**	**0.021**	**0.6**
**A14**	**0.06**	**1.2**
**A15**	**0.9**	**1.1**
**A3**	**0.9**	**0.9**
**A12**	**1.5**	**0.6**
**A6**	**6**	**1.1**
**A16**	**10**	**0.8**
**A4**	**10**	**1**
**A5**	**12**	**0.8**
**A11**	**13**	**0.9**
**A8**	**14**	**1.1**
**A13**	**17**	**0.7**
**A10**	**17**	**0.7**
**A9**	**17**	**1.4**
**A2**	**24**	**0.7**
**A1**	**NA**	**NA**
**A7**	**NA**	**NA**

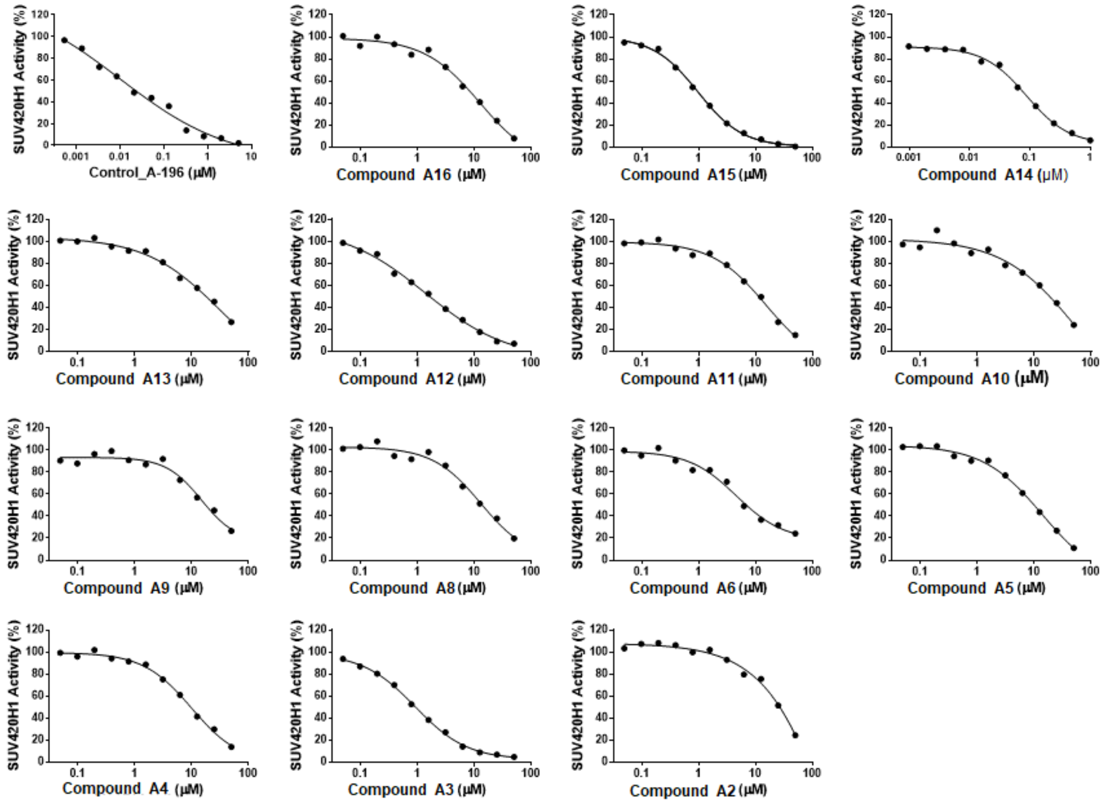

Supplementary Figure 8. IC_{50} determination of A-196 derivatives with SUV4-20 H1

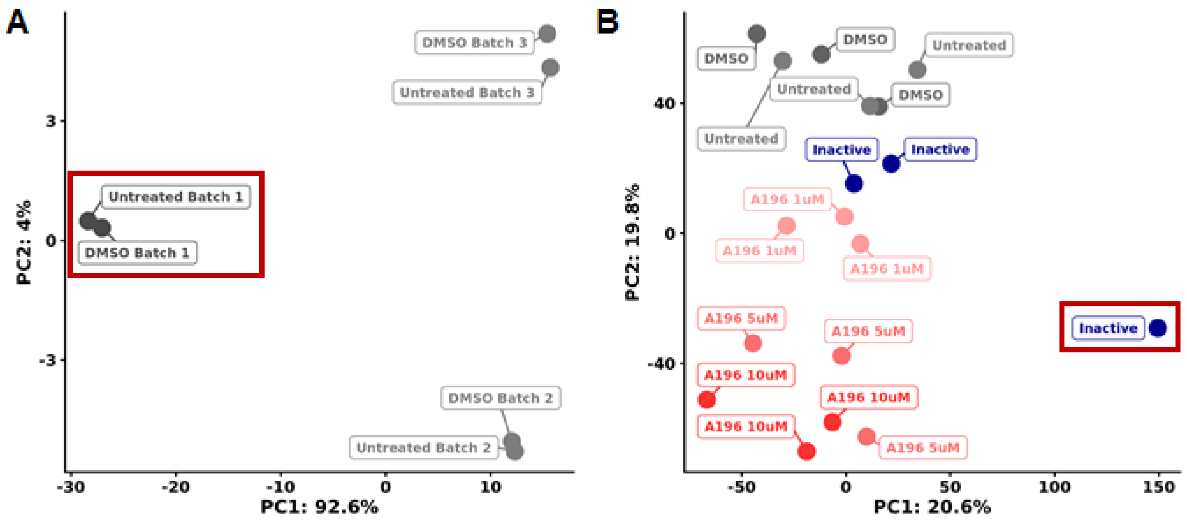

Supplementary Figure 9. PCA bi-plot of fibroblast lines GM034040 and GM04078 treated with A-196

Chemical structural modification of A-196

General Experimental

Solvents and reagents

All solvents were purchased from commercial sources and used without purification (HPLC or analytical grade). Anhydrous solvents were purchased from Acros Organics stored under a nitrogen atmosphere with activated molecular sieves. Standard vacuum line techniques were used and glassware was flame dried prior to use. Deionised water was sourced using an Elga DV 25 system. Organic solvents were dried during workup using anhydrous Na_2SO_4.

Purification and chromatography

Thin Layer Chromatography (TLC) was carried out using aluminium plates coated with 60 F254 silica gel. Plates were visualised using UV light (254 or 365 nm) or staining with Ninhydrin (1 M, EtOH) or 1% aq. $KMnO_4$. Normal-phase silica gel chromatography was carried out using Biotage Isolera One flash column chromatography system (LPLC).

Characterisation

NMR spectra were recorded using a Bruker Avance 400 MHz spectrometer using the deuterated solvent stated. Chemical shifts (δ) quoted in parts per million (ppm) and referenced to the residual solvent peak. Multiplicities are denoted as s - singlet, d - doublet, t - triplet, q - quartet, quin - quintet, m - multiplet and derivatives thereof (br denotes a broad resonance peak). Coupling constants recorded as Hz and round to the nearest 0.1 Hz. Two-dimensional NMR experiments (COSY, HSQC, HMBC) were used to aid the assignment of 1H and 13C spectra. Low Resolution mass spectra were recorded on a Waters SQ Detector 2 (LC-MS). Compound names were generated using ChemBioDraw Ultra v14 systematic naming.

Chemical Synthesis

Synthesis of 1,4,6,7-tetrachlorophthalazine

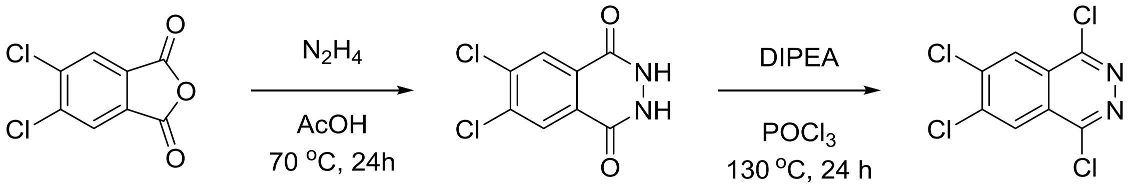

To a solution of 5,6-dichloroisobenzofuran-1,3-dione (5 g, 23.04 mmol) in acetic acid (20 ml) at room temp was added hydrazine (0.997 ml, 20.32 mmol) dropwise. The solution was stirred overnight at 70 °C under nitrogen. The excess acetic acid was removed *in vacuo* and the resultant 6,7-dichloro-2,3-dihydrophthalazine-1,4-dione (5.32 g, 23.03 mmol, quantitative) was dissolved in $POCl_3$ (21.46 ml, 230 mmol) immediately without further purification. N-ethyl-N-isopropylpropan-2-amine (4.01 ml, 23.03 mmol) was added dropwise and the mixture was heated overnight at 130 °C.

The reaction mixture was poured into ice water (400 mL) and left stirring for one hour. The crude product was extracted using dichloromethane (DCM, 3 x 300 mL) and washed with saturated sodium bicarbonate solution (5 x 100 mL) until pH = 7, followed by brine (3 x 50 mL). The combined organic extracts were dried over sodium sulfate and the excess solvent removed *in vacuo*. The crude product was purified using column chromatography (cyclohexane:EtOAc, 9:1) to yield 1,4,6,7-tetrachlorophthalazine (3.8 g, 14.18 mmol, 61.6 % yield) as white, crystalline solid. ^{1}H NMR (400 MHz, $CDCl_3$) δ 8.42 (s, 2H); ^{13}C NMR (101 MHz, $CDCl_3$) δ 153.29, 140.10, 127.23, 125.90; LRMS (ESI+) m/z 266.88

General Procedure A

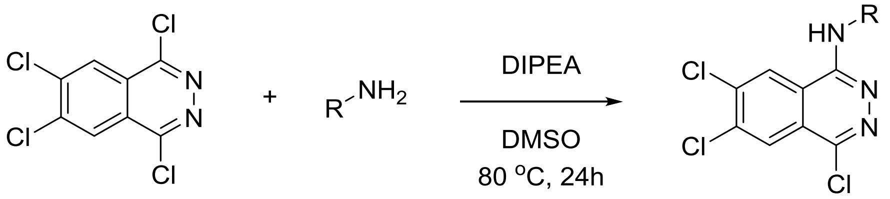

To a solution of 1,4,6,7-tetrachlorophthalazine (1 equiv.) and DIPEA (1.2 equiv) in DMSO (1 mL) in a 22 mL vial at 80 °C was added amine (1 equiv.) in DMSO (1 mL). The reaction was stirred at 80 °C overnight. Upon completion (as determined by LCMS), the reaction was cooled to room temperature and extracted with DCM (3 x 10 mL). The combined organic extracts were washed with water (5 x 10 mL), dried over sodium sulfate and the excess solvent removed *in vacuo*. The crude product was purified using column chromatography (cyclohexane:EtOAc, 9:1 to 7:3) to yield the desired compound.

4,6,7-trichloro-N-cyclopentylphthalazin-1-amine (A)

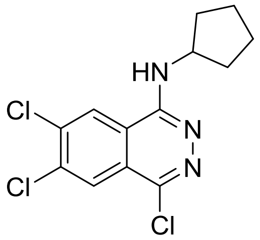

1,4,6,7-tetrachlorophthalazine (100 mg, 0.373 mmol) was reacted with cyclopentanamine (0.033 mL, 0.336 mmol) according to **general procedure A** to yield 4,6,7-trichloro-N-cyclopentylphthalazin-1-amine (81.3 mg, 0.257 mmol, 68.8 % yield) as a yellow solid. ^{1}H NMR (400 MHz, Chloroform-*d*) δ 8.25 (s, 1H), 7.85 (s, 1H), 5.01 (d, *J* = 6.5 Hz, 1H), 4.62 (q, *J* = 6.8 Hz, 1H), 2.29-2.21 (m, 2H), 1.83 – 1.65 (m, 4H), 1.61 – 1.51 (m, 2H); ^{13}C NMR (101 MHz, $CDCl_3$) δ 151.87, 143.83, 137.10, 136.76, 127.23, 125.28, 122.77, 118.84, 53.45, 33.16, 23.78; LRMS (ESI+) m/z 316.07

4,6,7-trichloro-N-(oxetan-3-yl)phthalazin-1-amine (B)

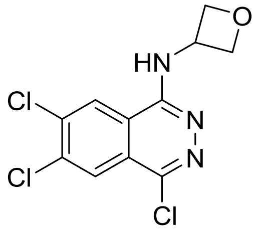

1,4,6,7-tetrachlorophthalazine (100 mg, 0.373 mmol) was reacted with oxetan-3-amine (27.3 mg, 0.373 mmol) according to **general procedure A** to yield 4,6,7-trichloro-N-(oxetan-3-yl)phthalazin-1-amine (75mg, 0.246 mmol, 66.0 % yield) as a yellow solid. ^{1}H NMR (400 MHz, Chloroform-*d*) δ 8.28 (s, 1H), 7.65 (s, 1H), 5.44 (s, 1H), 4.44 – 4.04 (m, 4H), 3.70 (dd, *J* = 12.4, 2.1 Hz, 1H); ^{13}C NMR

(101 MHz, $CDCl_3$) δ 151.56, 137.78, 137.36, 132.88, 127.31, 126.84, 125.54, 123.19, 64.23, 63.13, 51.95; LRMS (ESI+) m/z 303.99

4,6,7-trichloro-N-(2,3-dihydro-1H-inden-2-yl)phthalazin-1-amine (C)

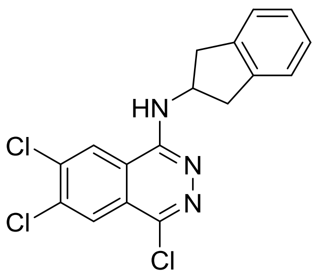

1,4,6,7-tetrachlorophthalazine (100 mg, 0.373 mmol) was reacted with 2,3-dihydro-1H-inden-2-amine (49.7 mg, 0.373 mmol) according to **general procedure A** to yield 4,6,7-trichloro-N-(2,3-dihydro-1H-inden-2-yl)phthalazin-1-amine (87 mg, 0.239 mmol, 64 % yield). ^{1}H NMR (400 MHz, Chloroform-*d*) δ 8.26 (s, 1H), 7.83 (s, 1H), 7.30-7.17 (m, 4H), 5.28 (d, *J* = 7.0 Hz, 1H), 5.16 (dt, *J* = 7.0, 4.0 Hz, 1H), 3.54 (dd, *J* = 16.4, 6.9 Hz, 2H), 3.03 (dd, *J* = 16.4, 3.9 Hz, 2H); ^{13}C NMR (101 MHz, $CDCl_3$) δ 151.79, 144.35, 140.88, 137.29, 136.92, 127.21, 126.68, 125.33, 124.84, 122.96, 118.85, 52.78, 40.08; LRMS (ESI+) m/z 364.07

4,6,7-trichloro-N-(2-(pyrrolidin-1-yl)ethyl)phthalazin-1-amine (D)

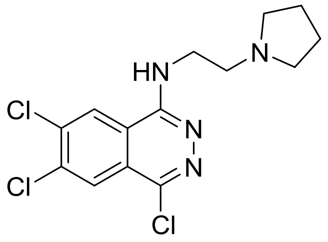

1,4,6,7-tetrachlorophthalazine (100 mg, 0.373 mmol) was reacted with 2-(pyrrolidin-1-yl)ethan-1-amine (0.047 ml, 0.373 mmol) according to **general procedure A** and the product eluted from the column using DCM:7M NH_3 in MeOH (90:10) to yield 4,6,7-trichloro-N-(2-(pyrrolidin-1-yl)ethyl)phthalazin-1-amine (93 mg, 0.134 mmol, 72 % yield) as a yellow solid. ^{1}H NMR (400 MHz, DMSO-d_6) δ 8.69 (s, 1H), 8.22 (s, 1H), 7.95 (t, *J* = 5.2 Hz, 1H), 3.54 (td, *J* = 7.0, 5.2 Hz, 2H), 2.44 (td, *J* = 4.7, 4.0, 2.1 Hz, 4H), 1.91 – 1.79 (m, 2H), 1.77 – 1.63 (m, 4H); ^{13}C NMR (101 MHz, $CDCl_3$) δ 154.93, 144.95, 137.96, 137.87, 126.78, 125.81, 125.56, 119.75, 55.50, 54.64, 38.34, 23.49; LRMS (ESI+) m/z 345.09

4,6,7-trichloro-N-((1-ethylpyrrolidin-2-yl)methyl)phthalazin-1-amine (E)

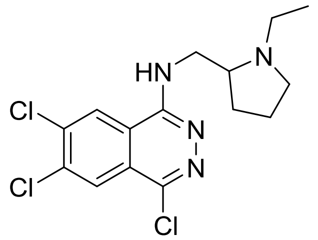

1,4,6,7-tetrachlorophthalazine (100 mg, 0.373 mmol) was reacted with (1-ethylpyrrolidin-2-yl)methanamine (0.054 ml, 0.373 mmol) according to **general procedure A** and the product eluted from the column using DCM:7M NH_3 in MeOH (90:10) to yield 4,6,7-trichloro-N-((1-ethylpyrrolidin-2-yl)methyl)phthalazin-1-amine (98 mg, 0.272 mmol, 73 % yield) as a white solid. ^{1}H NMR (400 MHz, Chloroform-*d*) δ 8.23 (s, 1H), 8.11 (s, 1H), 6.83 (s, 1H), 3.97 – 3.84 (m, 1H), 3.68 (dd, *J* = 13.9, 4.0 Hz, 1H), 3.45-3.41 (m, 1H), 3.19 – 3.06 (m, 1H), 2.98-2.89 (m, 2H), 2.51-2.41 (m, 2H), 1.83 – 1.72 (m, 3H), 1.19 (t, *J* = 7.2 Hz, 3H); ^{13}C NMR (101 MHz, $CDCl_3$) δ 153.15, 144.15, 137.63, 137.36, 127.29, 125.59, 124.01, 119.50, 63.42, 60.53, 53.77, 49.08, 42.22, 28.44, 23.24; LRMS (ESI+) m/z 359.10

4,6,7-trichloro-N-(1,2,3,4-tetrahydronaphthalen-1-yl)phthalazin-1-amine (F)

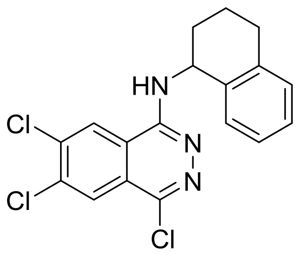

1,4,6,7-tetrachlorophthalazine (100 mg, 0.373 mmol) was reacted with 1,2,3,4-tetrahydronaphthalen-1-amine (0.054 ml, 0.373 mmol) according to **general procedure A** to yield 4,6,7-trichloro-N-(1,2,3,4-tetrahydronaphthalen-1-yl)phthalazin-1-amine (88 mg, 0.231 mmol, 62 % yield) as a white solid. ^{1}H NMR (400 MHz, DMSO-d_6) δ 8.92 (s, 1H), 8.25 (s, 1H), 8.10 (d, *J* = 8.1 Hz, 1H), 7.28 – 7.10 (m, 4H), 5.66 (d, *J* = 6.5 Hz, 1H), 2.82 (dt, *J* = 12.8, 6.3 Hz, 2H), 2.11 – 1.89 (m, 3H), 1.87 – 1.72 (m, 1H); ^{13}C NMR (101 MHz, $CDCl_3$) δ 151.76, 144.34, 140.00, 138.26, 137.57, 137.24, 136.88, 129.48, 129.44, 127.73, 127.46, 126.49, 123.31, 119.14, 49.55, 29.53, 28.97, 19.86; LRMS (ESI+) m/z 378.08

tert-butyl (S)-3-((4,6,7-trichlorophthalazin-1-yl)amino)pyrrolidine-1-carboxylate (G)

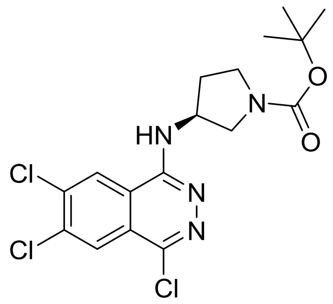

1,4,6,7-tetrachlorophthalazine (100 mg, 0.373 mmol) was reacted with tert-butyl (S)-3-aminopyrrolidine-1-carboxylate (63.3 µl, 0.373 mmol) according to **general procedure A** to yield tert-butyl (S)-3-((4,6,7-trichlorophthalazin-1-yl)amino)pyrrolidine-1-carboxylate (139 mg, 0.332 mmol, 89 % yield) as a white solid. ^{1}H NMR (400 MHz, Chloroform-*d*) δ 8.26 (d, *J* = 30.9 Hz, 2H), 6.11 (d, *J* = 106.0 Hz, 1H), 4.87 (s, 1H), 3.89 – 3.66 (m, 1H), 3.68 – 3.28 (m, 3H), 2.42 – 2.08 (m, 2H), 1.56 – 1.33 (m, 9H); ^{13}C NMR (101 MHz, $CDCl_3$) δ 154.97, 152.39, 144.75, 137.65, 137.28, 127.29, 125.65, 124.04, 119.36, 79.94, 53.56, 52.11, 44.54, 31.76, 28.71; LRMS (ESI+) m/z 417.10

4,6,7-trichloro-N-(2-morpholinoethyl)phthalazin-1-amine (H)

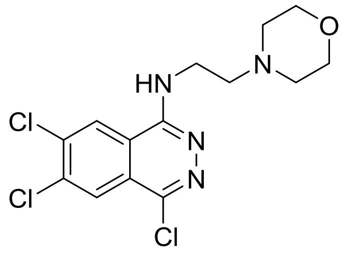

1,4,6,7-tetrachlorophthalazine (100 mg, 0.373 mmol) was reacted with 2-morpholinoethan-1-amine (48.6 mg, 0.373 mmol) according to **general procedure A** to yield 4,6,7-trichloro-N-(2-morpholinoethyl)phthalazin-1-amine (88 mg, 0.243 mmol, 65 % yield) as an off-white solid. ^{1}H NMR (400 MHz, Methanol-d_4) δ 8.38 (s, 1H), 8.13 (s, 1H), 4.46 (s, 1H), 3.69 – 3.58 (m, 6H), 2.65 (t, *J* = 6.5 Hz, 2H), 2.53 – 2.44 (m, 4H); ^{13}C NMR (101 MHz, MeOD) δ 154.91, 144.40, 138.63, 138.33, 128.05, 126.95, 126.46, 121.23, 67.87, 58.25, 54.97, 39.55; LRMS (ESI+) m/z 361.11

6,7-dichloro-N-(3-morpholinobutan-2-yl)-4-(pyridin-4-yl)phthalazin-1-amine (I)

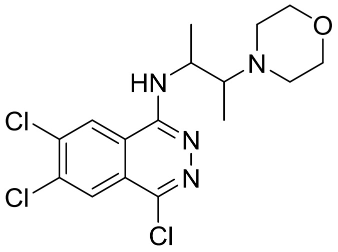

1,4,6,7-tetrachlorophthalazine (100 mg, 0.373 mmol) was reacted with 3-morpholinobutan-2-amine (0.058 ml, 0.373 mmol) according to **general procedure A** to yield 4,6,7-trichloro-N-(3-morpholinobutan-2-yl)phthalazin-1-amine (95 mg, 0.243 mmol, 65 % yield) as an off-white solid. ^{1}H NMR (400 MHz, Chloroform-*d*) δ 8.26 (s, 1H), 7.95 (s, 1H), 7.06 (s, 1H), 3.84-3.77 (m, 1H), 3.73-3.68 (m, 2H), 3.61 (br, 2H), 2.72-2.65 (m, 1H), 2.56 (br, 2H), 2.50 (br, 2H), 1.44 (d, *J* = 5.9 Hz, 3H), 1.14 (d, *J* = 6.6 Hz, 3H). ^{13}C NMR (101 MHz, $CDCl_3$) δ 166.90, 152.88, 137.45, 137.29, 127.58, 125.76, 123.30, 120.15, 67.83, 64.12, 49.65, 48.19, 18.27, 9.37; LRMS (ESI+) m/z 389.10

tert-butyl 3-((4,6,7-trichlorophthalazin-1-yl)amino)piperidine-1-carboxylate (J)

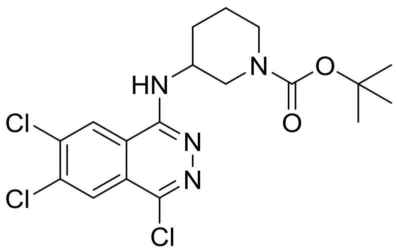

1,4,6,7-tetrachlorophthalazine (100 mg, 0.373 mmol) was reacted with tert-butyl 3-aminopiperidine-1-carboxylate (0.073 ml, 0.373 mmol) according to **general procedure A** to yield tert-butyl 3-((4,6,7-trichlorophthalazin-1-yl)amino)piperidine-1-carboxylate (118 mg, 0.272 mmol, 73 % yield) as a white solid. ^{1}H NMR (400 MHz, Chloroform-*d*) δ 8.23 (s, 1H), 7.93 (s, 1H), 6.33 (br, 1H), 4.40 (br, 1H), 4.26 – 2.99 (br m, 6H), 2.56 – 1.93 (br m, 2H), 1.50 (b, 9H); ^{13}C NMR (101 MHz, $CDCl_3$) δ 152.01, 144.19, 137.61, 137.36, 127.43, 125.65, 123.50, 119.32, 80.53, 48.86, 45.17, 44.32, 42.26, 28.61, 22.17; LRMS (ESI+) m/z 431.10

4,6,7-trichloro-N-(tetrahydro-2H-pyran-4-yl)phthalazin-1-amine (K)

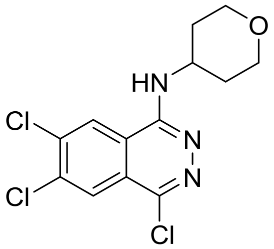

1,4,6,7-tetrachlorophthalazine (100 mg, 0.373 mmol) was reacted with tetrahydro-2H-pyran-4-amine (37.8 mg, 0.373 mmol) according to **general procedure A** to yield 4,6,7-trichloro-N-(tetrahydro-2H-pyran-4-yl)phthalazin-1-amine (98 mg, 0.295 mmol, 79 % yield) as a white solid. ^{1}H NMR (400 MHz, Chloroform-*d*) δ 8.28 (s, 1H), 7.88 (s, 1H), 4.90 (d, *J* = 7.2 Hz, 1H), 4.55-4.46 (m, 1H), 4.16 – 3.94 (m, 2H), 3.59 (td, *J* = 11.8, 2.1 Hz, 2H), 2.22 (ddd, *J* = 12.3, 4.3, 2.1 Hz, 2H), 1.69 – 1.58 (m, 2H); ^{13}C NMR (101 MHz, $CDCl_3$) δ 151.70, 144.52, 137.70, 137.34, 127.58, 125.73, 123.19, 119.11, 67.06, 48.10, 33.18; LRMS (ESI+) m/z 332.07

4,6,7-trichloro-N-cyclohexylphthalazin-1-amine (L)

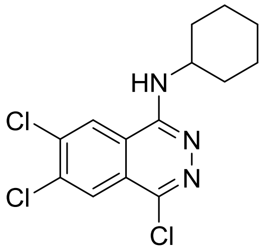

1,4,6,7-tetrachlorophthalazine (100 mg, 0.373 mmol) was reacted with cyclohexanamine (37.0 mg, 0.373 mmol) according to **general procedure A** to yield 4,6,7-trichloro-N-cyclohexylphthalazin-1-amine (101 mg, 0.306 mmol, 82 % yield) as a yellow solid. ^{1}H NMR (400 MHz, Chloroform-*d*) δ 8.16 (s, 1H), 7.83 (s, 1H), 4.97 (d, *J* = 7.5 Hz, 1H), 4.24-4.15 (m, 1H), 2.13 (dd, *J* = 12.0, 3.8 Hz, 2H), 1.71 (dt, *J* = 13.9, 3.8 Hz, 2H), 1.47 – 1.30 (m, 2H), 1.26-1.11 (m, 4H); ^{13}C NMR (101 MHz, $CDCl_3$) δ 151.80, 143.82, 137.38, 137.00, 127.43, 125.71, 123.25, 119.18, 50.51, 33.09, 25.89, 25.11; LRMS (ESI+) m/z 330.11

4,6,7-trichloro-N-((4-methylmorpholin-3-yl)methyl)phthalazin-1-amine (M)

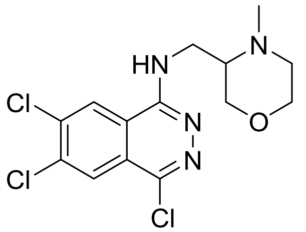

1,4,6,7-tetrachlorophthalazine (100 mg, 0.373 mmol) was reacted with (4-methylmorpholin-3-yl)methanamine (48.6 mg, 0.373 mmol) according to **general procedure B** to yield 4,6,7-trichloro-N-

((4-methylmorpholin-3-yl)methyl)phthalazin-1-amine (97 mg, 0.269 mmol, 72 % yield) as a white solid. ^{1}H NMR (400 MHz, Chloroform-*d*) δ 8.22 (s, 1H), 7.97 (s, 1H), 6.20 (s, 1H), 3.90 – 3.80 (m, 2H), 3.77 (d, *J* = 14.4 Hz, 1H), 3.71 – 3.61 (m, 2H), 3.52 (t, *J* = 11.8 Hz, 1H), 2.82 (dt, *J* = 11.8, 2.3 Hz, 1H), 2.59 – 2.53 (m, 1H), 2.52 – 2.43 (m, 1H), 2.39 (s, 3H); ^{13}C NMR (101 MHz, $CDCl_3$) δ 152.72, 144.31, 137.53, 137.15, 127.36, 125.48, 123.54, 119.26, 69.26, 66.93, 60.07, 54.95, 42.66, 39.78; LRMS (ESI+) m/z 361.08

4,6,7-trichloro-N-(tetrahydrofuran-3-yl)phthalazin-1-amine (N)

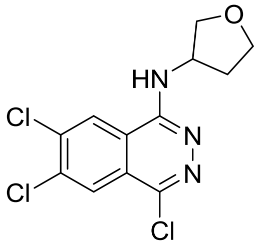

1,4,6,7-tetrachlorophthalazine (100 mg, 0.373 mmol) was reacted with tetrahydrofuran-3-amine (32.5 mg, 0.373 mmol) according to **general procedure A** to yield 4,6,7-trichloro-N-(tetrahydrofuran-3-yl)phthalazin-1-amine (94 mg, 0.295 mmol, 79 % yield) as a yellow solid. ^{1}H NMR (400 MHz, Chloroform-*d*) δ 8.28 (s, 1H), 7.89 (s, 1H), 5.22 (d, *J* = 6.6 Hz, 1H), 4.94 (ddt, *J* = 7.0, 4.6, 2.3 Hz, 1H), 4.07 (td, *J* = 8.1, 6.6 Hz, 1H), 4.00 (dd, *J* = 9.7, 5.1 Hz, 1H), 3.95 – 3.83 (m, 2H), 2.53-2.44 (m, 1H), 2.08 – 1.97 (m, 1H); ^{13}C NMR (101 MHz, $CDCl_3$) δ 151.61, 137.50, 137.19, 127.33, 125.31, 122.84, 118.82, 73.63, 66.86, 52.65, 33.16; LRMS (ESI+) m/z 318.01

4,6,7-trichloro-N-(3-(pyrrolidin-1-yl)propyl)phthalazin-1-amine (O)

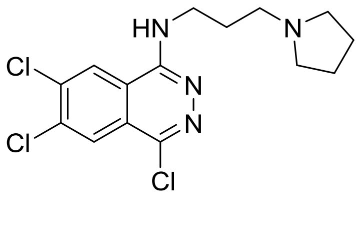

1,4,6,7-tetrachlorophthalazine (100 mg, 0.373 mmol) was reacted with 3-(pyrrolidin-1-yl)propan-1-amine (0.053 ml, 0.373 mmol) according to **general procedure A** and the product eluted from the column using DCM:7M NH_3 in MeOH (90:10) to yield 4,6,7-trichloro-N-(3-(pyrrolidin-1-yl)propyl)phthalazin-1-amine (110 mg, 0.306 mmol, 82 % yield) as an off-white solid. ^{1}H NMR (400 MHz, Chloroform-*d*) δ 8.92 (s, 1H), 8.18 (s, 1H), 7.80 (s, 1H), 3.77-3.73 (m, 2H), 2.91 – 2.78 (m, 2H), 2.78 – 2.64 (m, 4H), 2.05 – 1.97 (m, 4H), 1.94 (p, *J* = 5.6 Hz, 2H); ^{13}C NMR (101 MHz, $CDCl_3$)

δ 153.15, 143.12, 137.01, 136.70, 127.16, 125.57, 123.90, 119.82, 56.75, 54.42, 43.72, 25.06, 23.63; LRMS (ESI+) m/z 359.11

General Procedure B

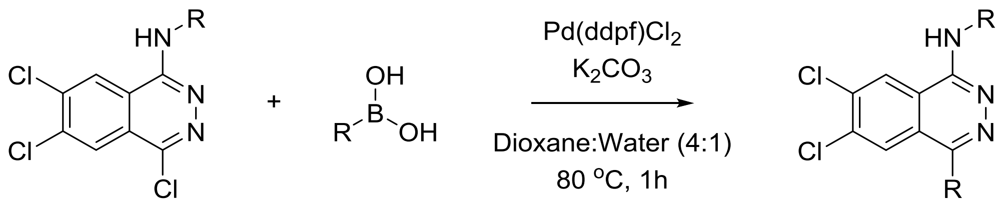

4,6,7-trichlorophthalazine-1-amine (1 equiv.), boronic acid (1 equiv.), $Pd(PPh_3)_4$ (0.1 equiv.), and K_2CO_3 (3 equiv.) were placed in a dry 22 mL vial equipped with stirrer bar. The vial was backfilled with N_2 and the contents dissolved in a degassed mixture of dioxane and water (4:1 ratio). The reaction was heated at 80 °C under N_2 for 3 hours before being cooled to room temperature and being filtered through celite. The mixture was extracted with ethyl acetate (3 x 10 mL) and the combined organic phases washed with water (3 x 10 mL) and brine (1 x 10 mL) before being dried over sodium sulfate. The residual solvent was removed *in vacuo* and the crude product purified using column chromatography (cyclohexane:ethyl acetate, 8:2 to 1:1).

6,7-dichloro-N-cyclopentyl-4-(pyrimidin-5-yl)phthalazin-1-amine (A1)

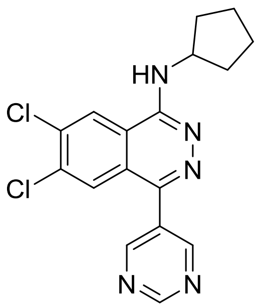

A (50 mg, 0.158 mmol) and pyrimidin-5-ylboronic acid (19.57 mg, 0.158 mmol) were reacted together according to **general procedure B** to yield 6,7-dichloro-N-cyclopentyl-4-(pyrimidin-5-yl)phthalazin-1-amine, compound **10** (40.4 mg, 0.112 mmol, 71 % yield) as a white solid. ^{1}H NMR (400 MHz, Chloroform-*d*) δ 9.35 (s, 1H), 9.10 (s, 2H), 7.98 (s, 1H), 7.95 (s, 1H), 5.26 (d, *J* = 6.5 Hz, 1H), 4.75 (q, *J* = 6.8 Hz, 1H), 2.37 – 2.24 (m, 2H), 1.87 – 1.68 (m, 4H), 1.64 – 1.56 (m, 2H); ^{13}C

NMR (101 MHz, $CDCl_3$) δ 158.74, 157.23, 151.97, 137.26, 136.47, 130.67, 126.71, 125.20, 123.36, 117.34, 53.87, 33.57, 24.12; LRMS (ESI+) m/z 360.34

6,7-dichloro-N-(oxetan-3-yl)-4-(pyridin-4-yl)phthalazin-1-amine (A2)

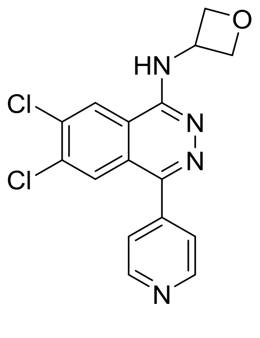

B (50 mg, 0.164 mmol) and pyridin-4-ylboronic acid (20.18 mg, 0.164 mmol) were reacted according to **general procedure B** to yield 6,7-dichloro-N-(oxetan-3-yl)-4-(pyridin-4-yl)phthalazin-1-amine, compound **11** (44.5 mg, 0.128 mmol, 78 % yield) as a yellow solid. ^{1}H NMR (400 MHz, Chloroform-*d*) δ 8.76 (dd, *J* = 4.4, 1.1 Hz, 2H), 8.39 (s, 1H), 7.49 (s, 1H), 7.41 (dd, *J* = 4.5, 1.6 Hz, 2H), 4.49 – 4.38 (m, 1H), 4.32 (d, *J* = 4.1 Hz, 1H), 4.11 (dd, *J* = 11.8, 3.1 Hz, 1H), 3.75 (dd, *J* = 11.8, 3.3 Hz, 1H); ^{13}C NMR (101 MHz, $CDCl_3$) δ 152.11, 150.62, 148.52, 142.16, 137.34, 136.38, 127.95, 127.30, 126.13, 123.91, 123.60, 64.44, 64.30, 52.93; LRMS (ESI+) m/z = 347.31

6,7-dichloro-N-(2,3-dihydro-1H-inden-2-yl)-4-(pyridin-4-yl)phthalazin-1-amine (A3)

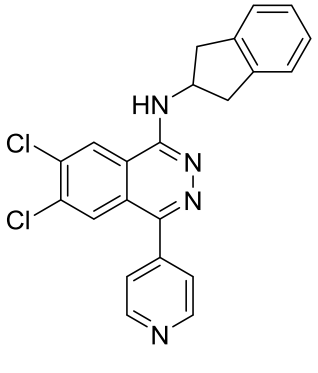

C (50 mg, 0.137 mmol) and pyridin-4-ylboronic acid (16.85 mg, 0.137 mmol) were reacted according to **general procedure B** to yield 6,7-dichloro-N-(2,3-dihydro-1H-inden-2-yl)-4-(pyridin-4-yl)phthalazin-1-amine, compound **12** (28 mg, 0.069 mmol, 50.1 % yield) as a white solid. ^{1}H NMR (400 MHz, Chloroform-*d*) δ 8.81 (d, *J* = 5.7 Hz, 2H), 8.01 (s, 1H), 7.90 (s, 1H), 7.63 (d, *J* = 6.0 Hz, 2H), 7.30 (dd, *J* = 5.4, 3.4 Hz, 2H), 7.25 – 7.20 (m, 2H), 5.38 (d, *J* = 7.0 Hz, 1H), 5.34 – 5.23 (m, 1H), 3.61 (dd, *J* = 16.4, 6.8 Hz, 2H), 3.09 (dd, *J* = 16.3, 3.9 Hz, 2H); ^{13}C NMR (101 MHz, $CDCl_3$) δ

151.80, 150.39, 149.20, 144.02, 141.25, 137.00, 136.31, 127.38, 127.04, 125.20, 125.02, 124.37, 123.21, 117.46, 53.16, 40.56; LRMS (ESI+) m/z 407.13

6,7-dichloro-4-(pyridin-4-yl)-N-(2-(pyrrolidin-1-yl)ethyl)phthalazin-1-amine (A4)

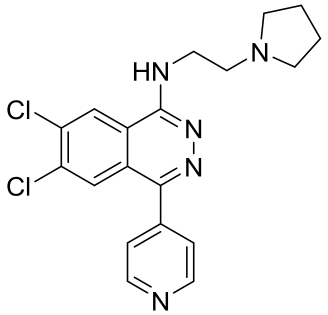

D (50 mg, 0.145 mmol) and pyridin-4-ylboronic acid (17.78 mg, 0.145 mmol) were reacted according to **general procedure B** and the product eluted from the column using DCM:7M NH_3 in MeOH (90:10) to yield 6,7-dichloro-4-(pyridin-4-yl)-N-(2-(pyrrolidin-1-yl)ethyl)phthalazin-1-amine, compound 14 (34.8 mg, 0.090 mmol, 62 % yield) as an off-white solid. ^{1}H NMR (400 MHz, Chloroform-*d*) δ 8.79 (dd, *J* = 2.8, 1.1 Hz, 2H), 8.45 (s, 1H), 7.95 (s, 1H), 7.60 (dd, *J* = 6.0, 2.8 Hz, 2H), 4.96 (br, 1H) 4.16 – 3.98 (m, 2H), 3.39 – 3.28 (m, 2H), 3.13 (d, *J* = 6.5 Hz, 4H), 2.05-1.98 (m, 4H); ^{13}C NMR (101 MHz, $CDCl_3$) δ 152.71, 150.32, 148.90, 144.25, 136.93, 136.31, 126.80, 125.14, 125.07, 124.32, 118.14, 54.65, 54.02, 38.83, 23.46; LRMS (ESI+) m/z 388.10

6,7-dichloro-N-((1-ethylpyrrolidin-2-yl)methyl)-4-(pyridin-4-yl)phthalazin-1-amine (A5)

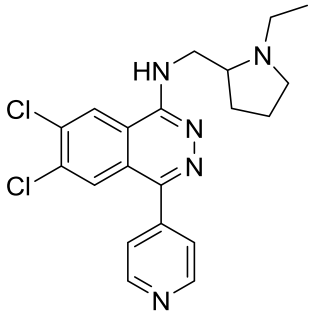

E (50 mg, 0.139 mmol) and pyridin-4-ylboronic acid (17.09 mg, 0.139 mmol) were reacted according to **general procedure B** and the product eluted from the column using DCM:7M NH_3 in MeOH (90:10) to yield 6,7-dichloro-N-((1-ethylpyrrolidin-2-yl)methyl)-4-(pyridin-4-yl)phthalazin-1-amine, compound **14** (34.7 mg, 0.086 mmol, 62 % yield) as a white solid. ^{1}H NMR (400 MHz, Chloroform-*d*) δ 8.81 (dd, *J* = 4.4, 1.5 Hz, 2H), 8.68 (s, 1H), 7.96 (s, 1H), 7.63 (dd, *J* = 4.4, 1.7 Hz, 2H), 6.88 (br, 1H), 4.11 – 4.01 (m, 1H), 3.95 (dd, *J* = 14.3, 9.7 Hz, 1H), 3.89 – 3.75 (m, 1H), 3.30 – 3.14 (m, 1H), 2.96 – 2.82 (m, 2H), 2.38 – 2.23 (m, 1H), 2.03 – 1.86 (m, 4H), 1.23 (t, *J* = 7.2 Hz, 3H); ^{13}C NMR

(101 MHz, $CDCl_3$) δ 152.77, 150.32, 149.45, 144.06, 137.62, 137.22, 126.81, 125.55, 125.10, 124.30, 119.43, 54.02, 52.01, 41.11, 29.83, 28.05, 25.81, 10.83; LRMS (ESI+) m/z 402.13

6,7-dichloro-4-(pyridin-4-yl)-N-(1,2,3,4-tetrahydronaphthalen-1-yl)phthalazin-1-amine (A6)

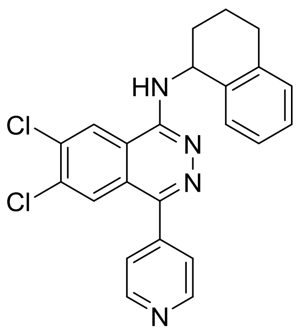

F (50 mg, 0.132 mmol) was reacted with pyridin-4-ylboronic acid (16.23 mg, 0.132 mmol) according to **general procedure B** to yield 6,7-dichloro-4-(pyridin-4-yl)-N-(1,2,3,4-tetrahydronaphthalen-1-yl)phthalazin-1-amine, compound **15** (37.3 mg, 0.088 mmol, 67 % yield) as an off-white solid. ^{1}H NMR (400 MHz, Chloroform-*d*) δ 8.82 (dd, *J* = 6.1, 1.5 Hz, 2H), 8.04 (s, 1H), 7.88 (s, 1H), 7.65 (dd, *J* = 4.5, 1.7 Hz, 2H), 7.41 (d, *J* = 7.6 Hz, 1H), 7.27 (s, 1H), 7.23 (q, *J* = 7.5, 6.6 Hz, 3H), 5.87 (q, *J* = 6.9, 5.6, 4.9 Hz, 1H), 5.37 (d, *J* = 7.5 Hz, 1H), 2.99 – 2.80 (m, 2H), 2.31 – 2.14 (m, 2H), 2.00 – 1.88 (m, 2H); ^{13}C NMR (101 MHz, $CDCl_3$) δ 151.35, 150.41, 149.12, 144.09, 138.36, 137.01, 136.99, 136.36, 129.62, 129.50, 127.85, 127.40, 126.62, 125.15, 124.36, 123.07, 117.35, 49.58, 29.58, 29.15, 19.88; LRMS (ESI+) m/z 421.10

tert-butyl (S)-3-((6,7-dichloro-4-(pyridin-4-yl)phthalazin-1-yl)amino)pyrrolidine-1-carboxylate (A7)

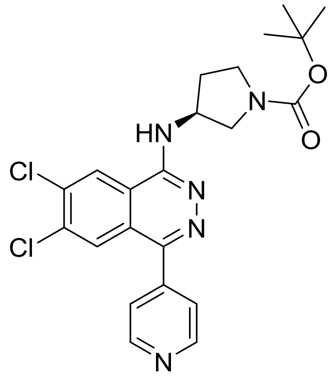

G (50 mg, 0.120 mmol) was reacted with pyridin-4-ylboronic acid (14.71 mg, 0.120 mmol) according to **general procedure B** to yield tert-butyl (R)-3-((6,7-dichloro-4-(pyridin-4-yl)phthalazin-1-yl)amino)pyrrolidine-1-carboxylate, compound **16** (29.8 mg, 0.065 mmol, 54 % yield) as a white solid. ^{1}H NMR (400 MHz, Chloroform-*d*) δ 8.81 (dd, *J* = 4.5, 1.4 Hz, 2H), 8.02 (s, 2H), 7.62 (dd, *J* = 4.5, 1.6 Hz, 2H), 5.48 (d, *J* = 63.5 Hz, 1H), 5.06 – 4.94 (m, 1H), 3.87 (d, *J* = 28.9 Hz, 1H), 3.70 – 3.37 (m, 3H), 2.51 – 2.33 (m, 1H), 2.28 – 2.12 (m, 1H), 1.46 (s, 9H); ^{13}C NMR (101 MHz, $CDCl_3$) δ

151.86, 150.32, 150.14, 149.40, 143.99, 137.15, 136.47, 127.32, 125.05, 124.34, 123.57, 79.97, 51.85, 44.49, 29.83, 28.69, 22.83, 14.26; LRMS (ESI+) m/z 460.16

6,7-dichloro-N-(2-morpholinoethyl)-4-(pyridin-4-yl)phthalazin-1-amine (A8)

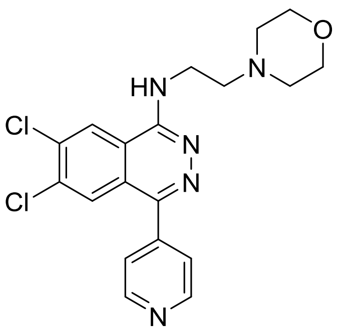

H (50 mg, 0.138 mmol) was reacted with pyridin-4-ylboronic acid (16.99 mg, 0.138 mmol) according to **general procedure B** to yield 6,7-dichloro-N-(2-morpholinoethyl)-4-(pyridin-4-yl)phthalazin-1-amine, compound **17** (43.0 mg, 0.106 mmol, 77 % yield) as a yellow solid. ^{1}H NMR (400 MHz, Chloroform-*d*) δ 8.77 (dd, *J* = 6.0, 1.6 Hz, 2H), 8.00 (d, *J* = 2.8 Hz, 2H), 7.56 (dd, *J* = 4.5, 1.6 Hz, 2H), 6.21 (br, 1H), 3.84 (t, *J* = 5.9 Hz, 2H), 3.78 (t, *J* = 4.7 Hz, 4H), 2.80 (t, *J* = 5.9 Hz, 2H), 2.59 (t, *J* = 5.8, 3.6 Hz, 5H); ^{13}C NMR (101 MHz, $CDCl_3$) δ 152.29, 150.35, 148.98, 144.08, 136.94, 136.27, 127.31, 124.96, 124.33, 123.40, 117.62, 67.11, 56.63, 53.40, 37.87; LRMS (ESI+) m/z 404.03

6,7-dichloro-N-(3-morpholinobutan-2-yl)-4-(pyridin-4-yl)phthalazin-1-amine (A9)

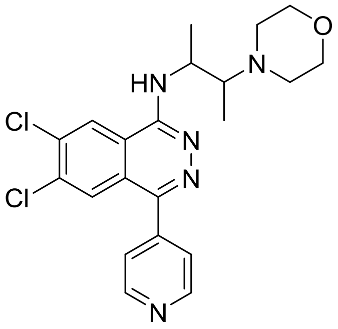

I (50 mg, 0.128 mmol) was reacted with pyridin-4-ylboronic acid (15.77 mg, 0.128 mmol) according to **general procedure B** to yield 6,7-dichloro-N-(3-morpholinobutan-2-yl)-4-(pyridin-4-yl)phthalazin-1-amine, compound **18** (36.1 mg, 0.083 mmol, 65 % yield) as a white solid. ^{1}H NMR (400 MHz, Chloroform-*d*) δ 8.81 (dd, *J* = 4.2, 1.7 Hz, 2H), 8.06 (s, 1H), 8.02 (s, 1H), 7.63 (dd, *J* = 4.4, 1.9 Hz, 2H), 4.02 – 3.88 (m, 1H), 3.82 – 3.56 (m, 4H), 2.82 – 2.68 (m, 1H), 2.67 – 2.45 (m, 4H), 1.51 (d, *J* = 5.9 Hz, 3H), 1.17 (d, *J* = 6.6 Hz, 3H); ^{13}C NMR (101 MHz, $CDCl_3$) δ 152.55, 150.30, 148.92, 136.86, 136.37, 132.30, 127.38, 125.07, 124.35, 123.36, 118.38, 67.80, 64.12, 55.18, 49.72, 18.49, 9.44; LRMS (ESI+) m/z 432.17

6,7-dichloro-N-(piperidin-3-yl)-4-(pyridin-4-yl)phthalazin-1-amine (A10)

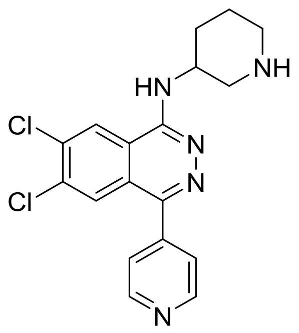

J (50 mg, 0.116mmol) was reacted with pyridin-4-ylboronic acid (14.24 mg, 0.116 mmol) according to **general procedure B** to yield tert-butyl 3-((6,7-dichloro-4-(pyridin-4-yl)phthalazin-1-yl)amino)piperidine-1-carboxylate (LRMS (ESI+) m/z 474.11). This was used directly without further purification and dissolved in dioxane (3 mL). HCl (4M in dioxane, 1 mL) was added and the solution left stirring overnight. The residual solvent was removed *in vacuo* and the product was re-dissolved in methanol (2 mL) before being applied to an Isolute SCX column that had been equilibrated with methanol. The column was washed with methanol (5 x 10mL) before the product was eluted with ammonia (7M in MeOH, 10 mL), to yield 6,7-dichloro-N-(piperidin-3-yl)-4-(pyridin-4-yl)phthalazin-1-amine, compound **19** (22.11 mg, 0.059 mmol, 51 % yield) as an off-white solid. ^{1}H NMR (400 MHz, Chloroform-*d*) δ 8.86 (s, 1H), 8.80 (dd, *J* = 4.4, 1.6 Hz, 2H), 7.97 (s, 1H), 7.60 (dd, *J* = 4.3, 1.9 Hz, 2H), 5.77 (s, 2H), 5.10 (s, 1H), 3.56 – 3.48 (m, 1H), 3.43 (d, *J* = 12.4 Hz, 1H), 3.25 (dd, *J* = 13.3, 3.6 Hz, 1H), 2.99 – 2.86 (m, 1H), 2.40 – 2.27 (m, 1H), 2.24 – 2.12 (m, 2H), 1.88 – 1.72 (m, 2H); ^{13}C NMR (101 MHz, $CDCl_3$) δ 152.03, 150.15, 149.05, 144.26, 137.06, 136.32, 126.85, 125.37, 125.24, 124.38, 118.16, 47.78, 44.04, 43.62, 26.52, 18.48; LRMS(ESI+) m/z 374.01

6,7-dichloro-4-(pyridin-4-yl)-N-(tetrahydro-2H-pyran-4-yl)phthalazin-1-amine (A11)

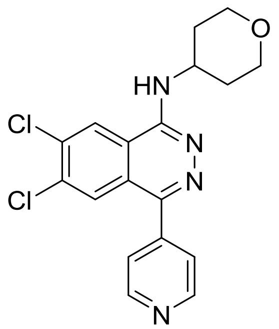

K (50 mg, 0.150 mmol) was reacted with pyridin-4-ylboronic acid (18.48 mg, 0.150 mmol) according to **general procedure B** to yield 6,7-dichloro-4-(pyridin-4-yl)-N-(tetrahydro-2H-pyran-4-yl)phthalazin-1-amine, compound **20** (35 mg, 0.093 mmol, 62.0 % yield) as a white solid. ^{1}H NMR (400 MHz, Chloroform-*d*) δ 8.80 (dd, *J* = 4.4, 1.3 Hz, 2H), 8.02 (s, 1H), 7.99 (s, 1H), 7.61 (dd, *J* = 4.5, 1.4 Hz, 2H), 5.14 (d, *J* = 7.2 Hz, 1H), 4.70 – 4.57 (m, 1H), 4.12 – 4.01 (m, 2H), 3.61 (td, *J* = 11.8,

2.0 Hz, 2H), 2.32 – 2.21 (m, 2H), 1.71 (dd, *J* = 11.7, 4.5 Hz, 2H); ^{13}C NMR (101 MHz, $CDCl_3$) δ 151.39, 150.36, 149.12, 143.98, 137.08, 136.40, 127.42, 125.09, 124.33, 123.12, 117.36, 67.12, 48.16, 33.32; LRMS (ESI+) m/z 375.17

6,7-dichloro-N-cyclohexyl-4-(pyridin-4-yl)phthalazin-1-amine (A12)

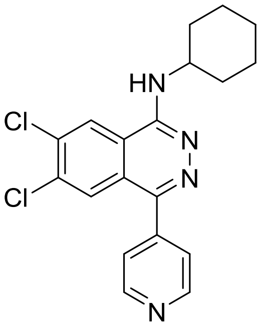

L (50 mg, 0.151 mmol) was reacted with pyridin-4-ylboronic acid (18.59 mg, 0.151 mmol) according to **general procedure B** to yield 6,7-dichloro-N-cyclohexyl-4-(pyridin-4-yl)phthalazin-1-amine, compound **21** (44.0 mg, 0.118 mmol, 78 % yield) as an off-white solid. ^{1}H NMR (400 MHz, Chloroform-*d*) δ 8.80 (dd, *J* = 4.3, 1.6 Hz, 2H), 8.02 (s, 1H), 7.92 (s, 1H), 7.62 (dd, *J* = 4.4, 1.6 Hz, 2H), 5.00 (d, *J* = 7.5 Hz, 1H), 4.48 – 4.35 (m, 1H), 2.34 – 2.22 (m, 3H), 1.89 – 1.78 (m, 2H), 1.52 – 1.45 (m, 2H), 1.40 – 1.27 (m, 4H); ^{13}C NMR (101 MHz, $CDCl_3$) δ 151.36, 150.36, 149.02 143.61, 136.87, 136.12, 126.57, 124.85, 124.35, 123.08, 118.03, 50.21, 32.72, 26.12, 25.05; LRMS (ESI+) m/z 373.11

6,7-dichloro-N-((4-methylmorpholin-3-yl)methyl)-4-(pyridin-4-yl)phthalazin-1-amine (A13)

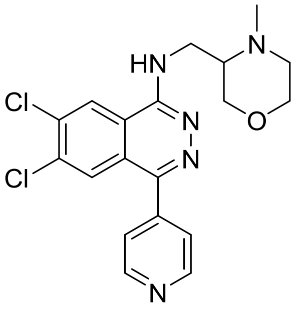

M (50 mg, 0.138 mmol) was reacted with pyridin-4-ylboronic acid (16.99 mg, 0.138 mmol) according to **general procedure B** to yield 6,7-dichloro-N-((4-methylmorpholin-3-yl)methyl)-4-(pyridin-4-yl)phthalazin-1-amine, compound **22** (36.3 mg, 0.090 mmol, 65 % yield) as a white solid. ^{1}H NMR (400 MHz, Chloroform-*d*) δ 8.80 (dd, *J* = 4.4, 1.6 Hz, 2H), 8.01 (d, *J* = 6.4 Hz, 2H), 7.60 (dd, *J* = 4.4,

1.5 Hz, 2H), 6.20 (s, 1H), 3.95 – 3.75 (m, 4H), 3.71 (td, *J* = 11.4, 2.4 Hz, 1H), 3.62 (dd, *J* = 11.7, 10.3 Hz, 1H), 2.87 (dt, *J* = 11.8, 2.2 Hz, 1H), 2.67 – 2.58 (m, 1H), 2.52 (td, *J* = 11.5, 3.4 Hz, 1H), 2.43 (s, 3H); ^{13}C NMR (101 MHz, $CDCl_3$) δ 152.40, 150.36, 149.14, 144.06, 137.04, 136.33, 127.37, 124.97, 124.35, 123.36, 117.59, 69.21, 66.98, 60.20, 55.05, 42.64, 39.73; LRMS (ESI+) m/z 404.08

6,7-dichloro-N-cyclopentyl-4-(1-methyl-1H-pyrazol-3-yl)phthalazin-1-amine (A14)

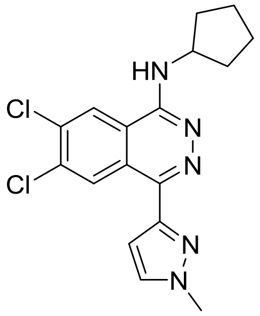

A (50 mg, 0.158 mmol) and 1-methyl-1H-pyrazole-3-boronic acid pinacol ester (32.9 mg, 0.158 mmol) were reacted according to **general procedure B** to yield 6,7-dichloro-N-cyclopentyl-4-(1-methyl-1H-pyrazol-3-yl)phthalazin-1-amine, compound **13** (31 mg, 0.086 mmol, 54.2 % yield) as a white solid. ^{1}H NMR (400 MHz, Chloroform-*d*) δ 9.56 (s, 1H), 7.85 (s, 1H), 7.44 (d, *J* = 2.3 Hz, 1H), 7.10 (d, *J* = 2.3 Hz, 1H), 5.02 (d, *J* = 6.4 Hz, 1H), 4.71 (h, *J* = 6.7 Hz, 1H), 4.04 (s, 3H), 2.34 – 2.22 (m, 2H), 1.86 – 1.66 (m, 5H), 1.66 – 1.52 (m, 4H); ^{13}C NMR (101 MHz, $CDCl_3$) δ 151.52, 150.40, 143.63, 136.25, 135.37, 130.94, 130.07, 124.97, 122.30, 117.77, 106.74, 53.68, 39.49, 33.63, 24.12; LRMS (ESI+) m/z 362.15.

6,7-dichloro-4-(pyridin-4-yl)-N-(tetrahydrofuran-3-yl)phthalazin-1-amine (A15)

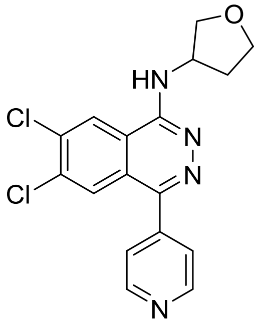

N (50 mg, 0.157 mmol) and pyridin-4-ylboronic acid (19.29 mg, 0.157 mmol) were reacted together according to **general procedure B** to yield 6,7-dichloro-4-(pyridin-4-yl)-N-(tetrahydrofuran-3-yl)phthalazin-1-amine, compound **24** (42.0 mg, 0.116 mmol, 74 % yield) as a white solid. ^{1}H NMR (400 MHz, Chloroform-*d*) δ 8.80 (dd, *J* = 4.4, 1.7 Hz, 2H), 8.01 (d, *J* = 5.8 Hz, 2H), 7.61 (dd, *J* = 4.4, 1.7 Hz, 2H), 5.44 (d, *J* = 6.7 Hz, 1H), 5.10 – 4.98 (m, 1H), 4.14 – 4.02 (m, 2H), 3.98 (dd, *J* = 9.7, 2.4 Hz, 1H), 3.90 (td, *J* = 8.7, 5.9 Hz, 1H), 2.58 – 2.47 (m, 1H), 2.15 – 2.04 (m, 1H); ^{13}C NMR (101 MHz, $CDCl_3$) δ 151.68, 150.39, 149.45, 143.91, 137.18, 136.56, 127.45, 125.00, 124.34, 123.23, 117.50, 74.07, 67.23, 53.00, 33.59; LRMS (ESI+) m/z 361.10

6,7-dichloro-4-(pyridin-4-yl)-N-(3-(pyrrolidin-1-yl)propyl)phthalazin-1-amine (A16)

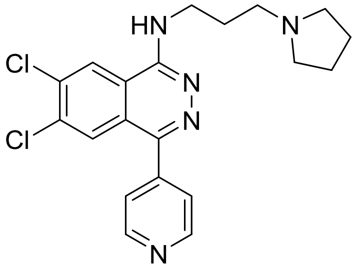

O (50 mg, 0.139 mmol) was reacted with pyridin-4-ylboronic acid (17.09 mg, 0.139 mmol) according to **general procedure B** and the product eluted from the column using DCM:7M NH_3 in MeOH (90:10) to yield 6,7-dichloro-4-(pyridin-4-yl)-N-(3-(pyrrolidin-1-yl)propyl)phthalazin-1-amine, compound **25** (30.2 mg, 0.075 mmol, 54 % yield) as an off white solid. ^{1}H NMR (400 MHz, Chloroform-*d*) δ 8.79 (dd, *J* = 4.5, 1.6 Hz, 2H), 8.19 (s, 1H), 7.95 (s, 1H), 7.61 (dd, *J* = 4.4, 1.7 Hz, 2H), 3.89 – 3.82 (m, 2H), 3.63-3.51 (m, 2H) 3.13-2.99 (m, 4H), 2.25-2.11 (m, 4H), 1.98-1.91 (m, 2H); ^{13}C NMR (101 MHz, $CDCl_3$) δ 152.43, 150.43, 149.03 142.92, 136.45, 136.02, 126.74, 125.02, 124.40 122.98, 119.03, 57.02, 55.11, 43.24, 25.75, 23.83; LRMS (ESI+) m/z 402.15
